# Supplementary material for: Mycobacterium tuberculosis-dependent monocyte expression quantitative trait loci, cytokine production, and TB pathogenesis
Source: Front Immunol. 2024 Mar 7;15:1359178. doi: 10.3389/fimmu.2024.1359178 (PMC10954790; doi:10.3389/fimmu.2024.1359178)
Supplement: Supplementary file 1 [file DataSheet_1.pdf]

Supplementary Material

1.1 Supplementary Figures

Figs. S1 to S6.

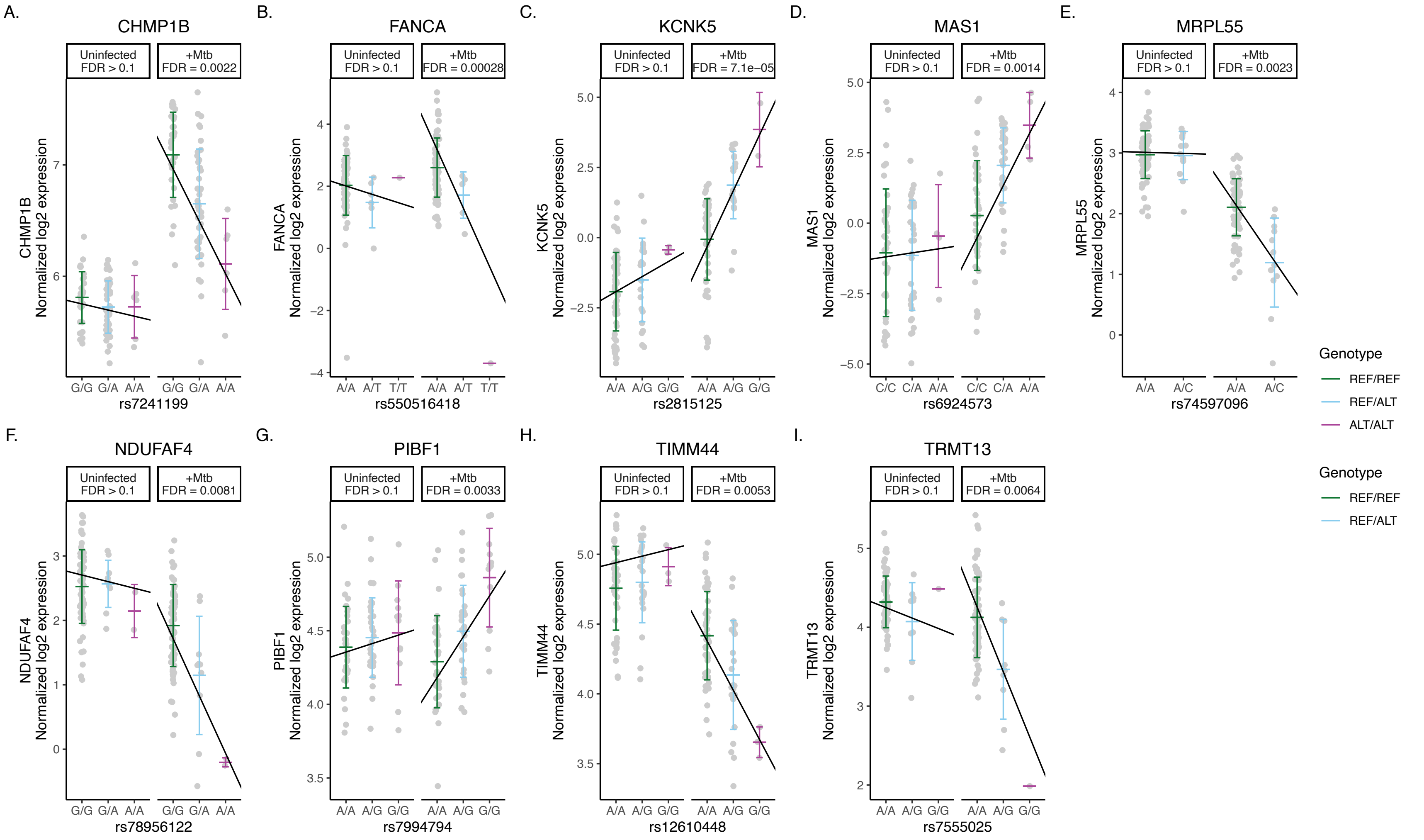

Figure S1. Expression plots for the remaining 9 Mtb-dependent eQTLs. This figure displays the association between 9 Mtb-dependent eQTLs and the expression of their target genes, which are not presented in the main text. The x-axis represents the different genotypes, while the y-axis shows the normalized log2 expression of target genes. The lines indicate a linear fit derived from an

additive regression model that includes an interaction term (Mtb_infection:Genotype), adjusting for age and sex. The FDR represents the significance level for eQTLs in either the Mtb-uninfected condition or the Mtb-infected condition, not the interaction model.

ALT= alternative allele, FDR= false discovery rate, REF= reference allele

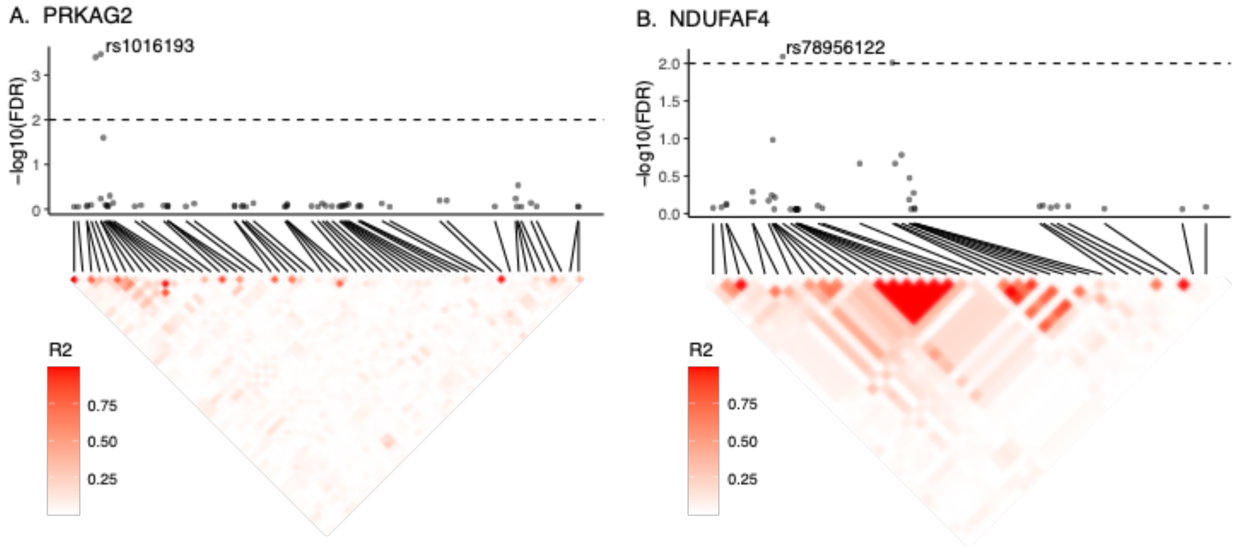

Figure S2. Pattern of linkage disequilibrium. *PRKAG2* and *NDUFAF4* had 2 cis eQTLs in high linkage disequilibrium (LD). (top) eQTL significance for SNPs within 1 MB of **(A)** *PRKAG2* and **(B)** *NDUFAF4*. X-axis indicates chromosome position of the eQTLs. Horizontal dashed line indicates FDR = 0.01 and the lead SNP is labeled. (bottom) Heatmap indicating R^2 LD for SNPs in this region.

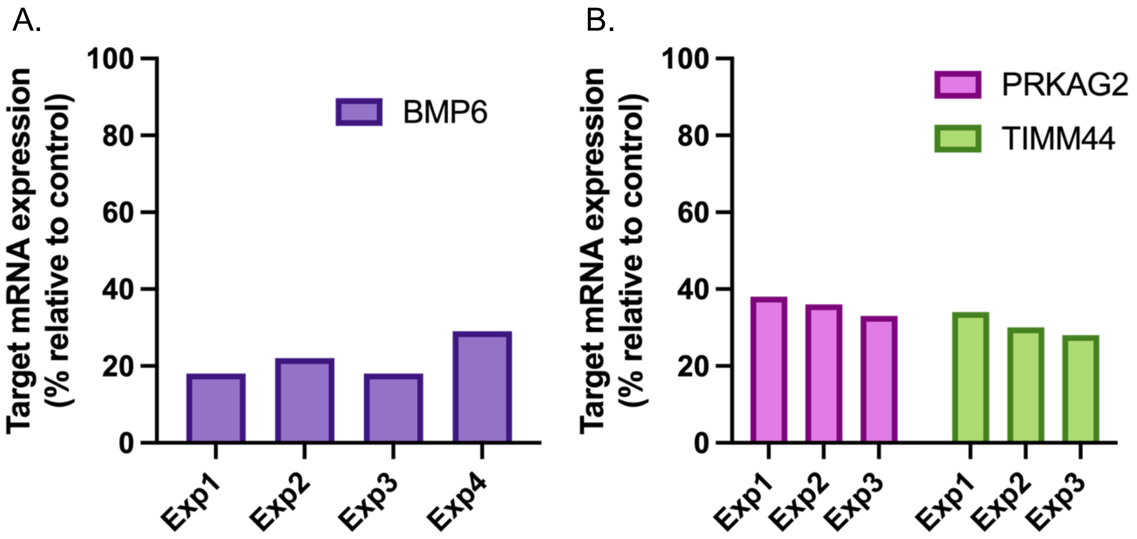

Figure S3. siRNA knockdown efficiency in THP-1 cells. qRT-PCR was performed to quantitate targeted mRNA levels in THP-1 cells treated with either scrambled control siRNA or target gene siRNA (*BMP6*, *PRKAG2*, and *TIMM44*). For the siRNA-treated cells from each experiment, targeted mRNA levels were normalized to *GAPDH* mRNA levels, and each sample was normalized to control siRNA-treated cells. (A) The range of % relative expression compared to siRNA control for *BMP6* ranged from 18% to 29%. (B) The range of % relative expression compared to siRNA control ranged from 33% to 38% for *PRKAG2* and from 28% to 34% for *TIMM44*.

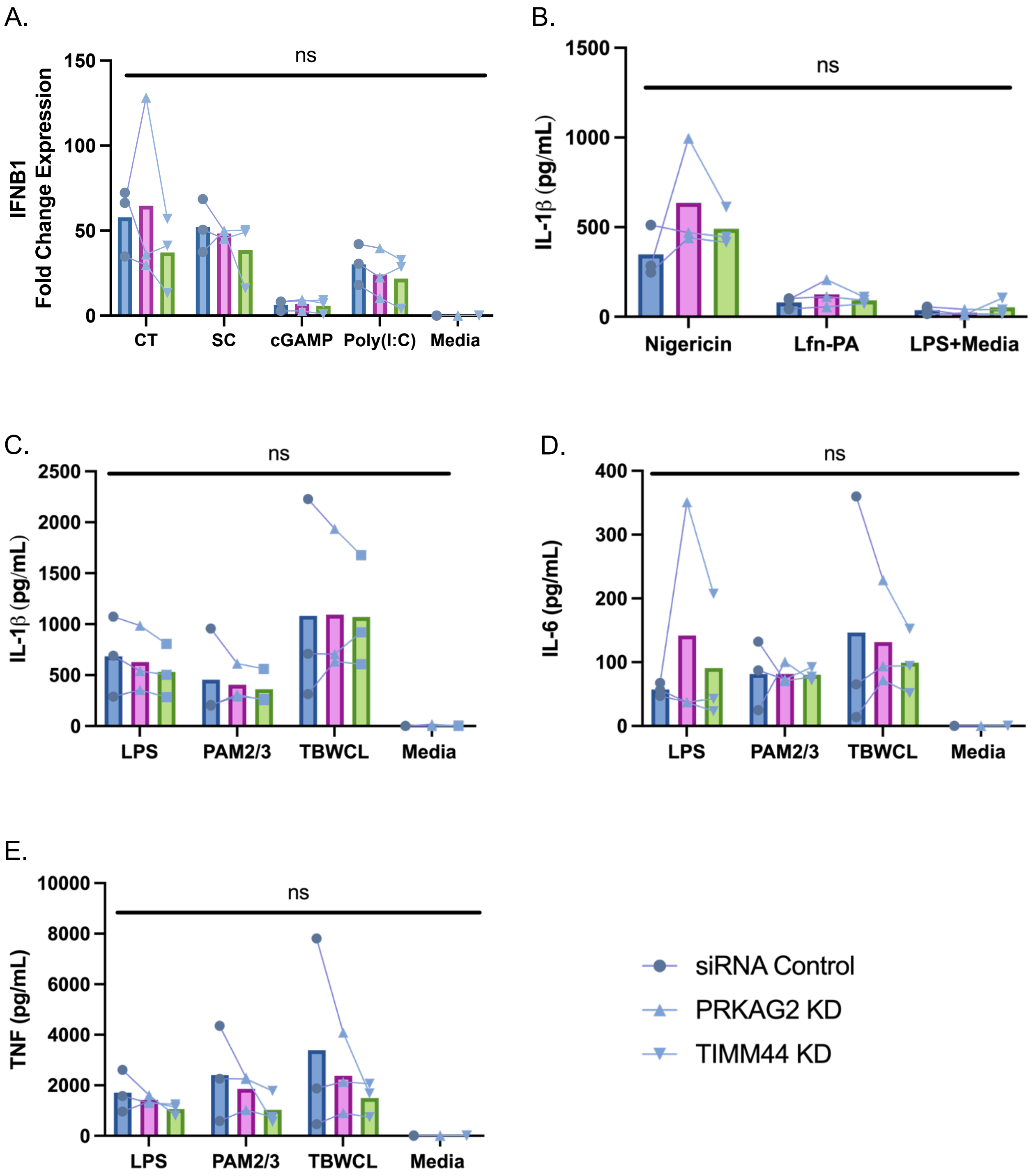

Figure S4. Cytokine responses in *PRKAG2*- and *TIMM44*-silenced THP-1 cells stimulated with DNA, TLR, and inflammasome ligands. *PRKAG2*-silenced and *TIMM44*-silenced THP-1 cells, when stimulated with various ligands (calf thymus DNA, supercoiled plasmid DNA, cGAMP, or poly(I:C)), showed no significant changes in **(A)** *IFNB1* expression compared to siRNA control cells. To evaluate the inflammasome-mediated IL-1β response, nucleofected cells were primed with LPS for 2h and then treated with nigericin for *NLRP3*-specific stimulation or

Burkholderia thailandensis needle protein with *Bacillus anthracis* protective antigen for *NLRC4*-specific stimulation. There were also no significant differences observed between *PRKAG2*-silenced or *TIMM44*-silenced cells and siRNA control cells in inflammasome-mediated **(B)** IL-1β response. For TLR-specific stimulation, *PRKAG2*-silenced, *TIMM44*-silenced, and siRNA control THP-1 cells were stimulated with LPS, PAM2/PAM3, Mtb whole cell lysate, or media. There were no significant differences in **(C)** IL-1β, **(D)** IL-6, and **(E)** TNF supernatant levels at 24h stimulation. *IFNB1* expression was quantified by qRT-PCR and normalized against background induction from lipofectamine. IL-1β, IL-6, and TNF supernatant levels were measured by ELISA.

cGAMP = cyclic guanosine monophosphate-adenosine monophosphate, CT = calf thymus DNA, KD = knockdown, LPS = lipopolysaccharide, media = media control (RPMI + 10% FBS), PAM2 = Pam2CSK4, PAM3 = Pam3CSK, poly(I:C) = polyinosinic acid-polycytidylic acid, SC = supercoiled plasmid DNA, TBWCL = Mtb whole cell lysates, TLR = toll-like receptor, ns= not significant.

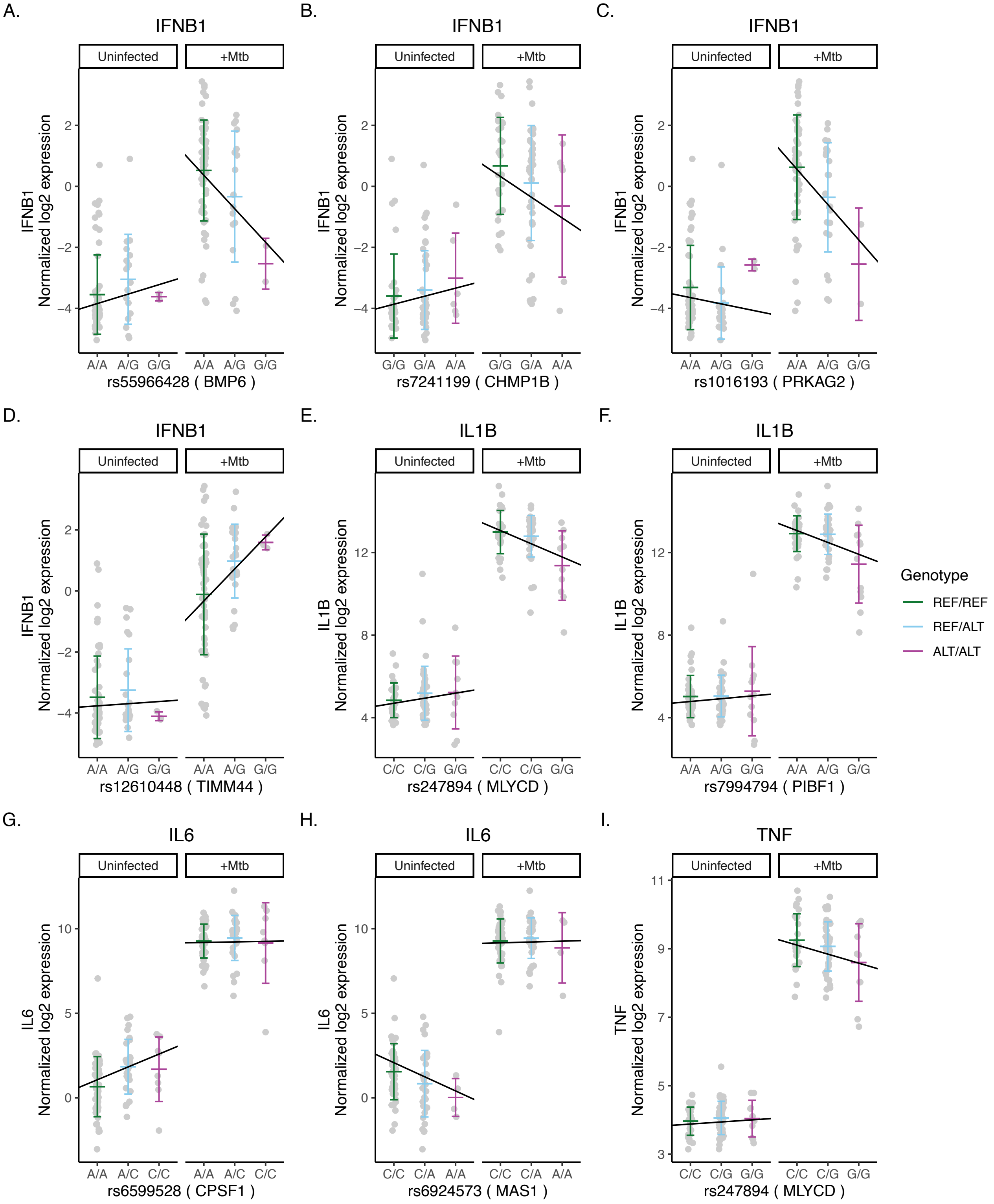

Figure S5. Mtb-dependent eQTLs associated with Mtb-induced cytokine expression in Mtb-infected and uninfected monocytes. While the relationship between host genotype and differences in cytokine expression was established through the subtraction of cytokine expression from Mtb-

infected to uninfected conditions (i.e., normalized log2 [Mtb-infected – uninfected relative cytokine expression]), this figure depicts the correlation between Mtb-dependent eQTLs and the expression of cytokine genes in Mtb-uninfected media and Mtb-infected conditions separately. The x-axis represents different genotypes, while the y-axis represents the normalized log2 expression of cytokine genes. The slope of the lines indicates the ratio of the effect estimate to the standard error derived from a linear regression model, which estimates the relationship between the number of minor alleles and the differences in cytokine expression in each condition, controlling for age and sex ($p < 0.05$).

ALT= alternative allele, REF= reference allele.

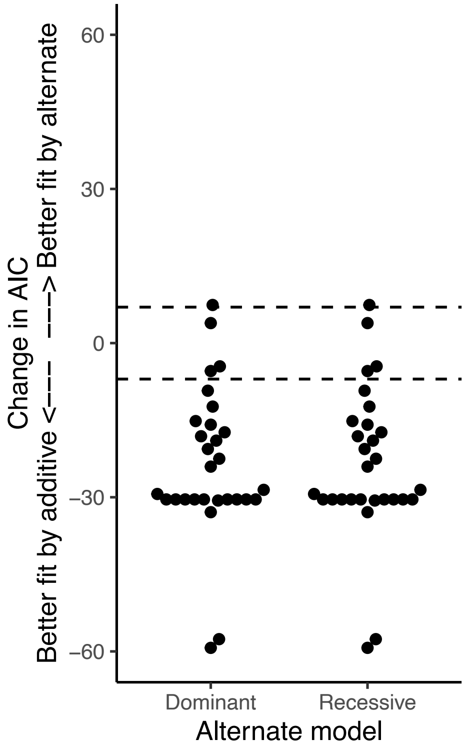

Figure S6. Comparison of genetic model fit: additive vs. dominant or recessive models. Model fit was measured by Akaike Information Criterion (AIC) for significant additive eQTLs. The change in AIC was calculated for each eQTL by comparing the additive model to the dominant or recessive model (i.e., AIC of the additive model minus AIC of the dominant/recessive model; a negative value indicates that the additive model is a better fit, and vice versa). Dashed lines indicate minimal change in AIC (-7 < delta AIC < 7). The change in AIC ranged from -59.31 to 7.41, with a median of -24.
